# Anatomical Considerations for Hyaluronic Acid Filler Injection for Breast Augmentation in Young Female Patients

**DOI:** 10.3390/life15040624

**Published:** 2025-04-08

**Authors:** Jae Hun Hur, Jovian Wan, Song Eun Yoon, Sky Wong, Kyu-Ho Yi

**Affiliations:** 1 247 Clinic, 108 Bongeunsa-ro, Gangnam District, Seoul, Republic of Korea; 2 Medical Research Inc., Wonju, Republic of Korea; 3 BRANDNEW Aesthetic Surgery Clinic, Seoul, Republic of Korea; 4 Leciel Medical Centre, Hong Kong; 5 Division in Anatomy and Developmental Biology, Department of Oral Biology, Human Identification Research Institute, BK21 FOUR Project, Yonsei University College of Dentistry, 50-1 Yonsei-ro, Seodaemun-gu, Seoul 03722, Republic of Korea; 6 You and I Clinic, Seoul 06001, Republic of Korea

**Keywords:** breast augmentation, hyaluronic acid, injections, subcutaneous, anatomy, cross-sectional, ultrasonography, interventional

## Abstract

Background: Hyaluronic acid (HA) fillers offer a minimally invasive alternative for breast augmentation, appealing to young female patients seeking natural results and minimal recovery time. However, achieving optimal outcomes requires a thorough understanding of breast anatomy, filler properties, and safe injection techniques. This study provides a comprehensive analysis of the anatomical considerations, techniques, and filler properties necessary for optimal breast augmentation using HA filler. It also explores patient selection, long-term safety and efficacy, and the management of complications. A review of key anatomical structures, including glandular tissue, fascial layers, vascular anatomy, and Cooper’s ligaments was conducted. Injection techniques, such as dual-plane and submuscular approaches, were analysed with a focus on pre-procedural imaging. Four cases of young female patients undergoing breast augmentation using HA filler (e.p.t.q. eve X, Jetema Inc., Seoul, Republic of Korea) were analysed. The efficacy of HA fillers in achieving natural breast enhancement was demonstrated in all four cases. Ultrasound played a crucial role in ensuring accurate filler placement, reducing risks like vascular occlusion and filler migration. Patients reported high satisfaction and minimal complications, though periodic treatments were required for maintenance. HA filler-based breast augmentation is a safe and effective option for young female patients, delivering natural results with rapid recovery. Although the results can be temporary and maintenance treatment is required, HA fillers may offer a non-invasive alternative to silicone implants. Optimal outcomes can be achieved through a thorough understanding of anatomy, the use of highly cohesive fillers, and ultrasound-guided injection techniques.

## 1. Introduction

Breast augmentation is one of the most frequently performed aesthetic procedures worldwide, and its popularity continues to grow, particularly among younger women [1,2,3]. Traditionally, silicone and saline implants have been the most common choices for this procedure [4,5,6]. However, recently, hyaluronic acid (HA) fillers have emerged as a non-surgical alternative, offering several advantages. These include shorter recovery times, the use of local anaesthesia, and the ability to achieve customisable results, allowing patients to enhance breast volume without invasive surgery [7,8,9]. Despite these benefits, the use of HA fillers in breast augmentation is not without risks. Complications such as infection, filler migration, asymmetry, and interference with breast cancer screening have raised concerns about the safety and long-term effectiveness of this technique [6,8]. For example, products like Macrolane, previously marketed for breast augmentation, were withdrawn due to issues with radiological imaging that could obscure breast cancer detection [10,11]. Furthermore, complications such as capsular contracture and nodule formation have been reported, particularly when larger volumes are used or when fillers are not injected correctly [12,13,14].

This article aims to explore the anatomical considerations, techniques, and filler properties required for safe and effective HA filler breast augmentation in young female patients. We also review documented complications and propose strategies for minimising risks while achieving natural-looking, aesthetically pleasing results.

### 1.1. Anatomical Considerations

Understanding the anatomy of the breast is essential when planning HA filler injections, as the breast’s structure is complex and variable. It is composed of glandular tissue, fat, and connective structures that collaborate to provide shape, support, and function. These components are further organised within a network of fascial planes, ligaments, and vascular structures that are critical for both the aesthetics and safety of breast augmentation procedures [15,16,17,18,19].

The glandular tissue, responsible for milk production, is arranged in lobules connected to ducts that converge toward the nipple. Surrounding this tissue are varying amounts of subcutaneous fat, contributing to the breast’s volume and shape. The ratio of glandular tissue to fat varies by age, hormonal status, and body composition. Younger and non-pregnant patients typically have firmer breasts with more glandular tissue and less fat, while older patients and those who have breastfed have more fat. This variation requires greater care in patients with more glandular tissue to prevent visible lumps or nodules, as denser tissue may make the filler more noticeable [18,19].

The pectoralis fascia separates the breast tissue from the underlying pectoralis major muscle, defining the boundary between the breast and chest wall. Above this fascia, the retromammary (submammary) space is commonly targeted for HA filler injections. This space allows the filler to integrate smoothly, providing better blending and coverage while preserving the natural movement of the breast. Proper placement in this space reduces the risk of surface irregularities and undesirable cosmetic outcomes such as visible filler or unnatural contours [19,20].

Cooper’s ligaments are fibrous connective tissues that extend from the chest wall to the skin, maintaining the structural integrity of the breast. These ligaments form a supportive framework that can influence the positioning and shape of the filler [21,22]. The tension and orientation of these ligaments are critical factors in achieving a natural contour when using HA fillers. Improper filler placement or disruption of these ligaments can result in uneven distribution and an unnatural appearance.

The breast lies primarily on the pectoralis major muscle, which contributes to the definition of the chest wall. Along with the serratus anterior muscle laterally, the pectoralis major provides a stable surface against which the breast sits [23,24]. Depending on the patient’s anatomy and the desired outcome, HA fillers can be injected either submuscularly (beneath the pectoralis major muscle) or subglandularly (above the pectoralis major but beneath the glandular tissue). Subglandular injection is usually preferred as it minimises interference with posttreatment breast imaging and palpation. Submuscular injections may be recommended for patients with minimal breast tissue, as the pectoralis muscle may provide additional coverage and help prevent visible lumps [11].

### 1.2. Neurovascular Anatomy

Vascular supply to the breast plays an essential role in ensuring safe HA filler injections. The breast is predominantly supplied by branches of the internal mammary artery and the lateral thoracic artery. The internal mammary artery runs along the medial aspect of the breast, while the lateral thoracic artery supplies the lateral and inferior portions. Smaller branches from the intercostal arteries and thoracoacromial artery also contribute to the vascular network. Understanding the anatomy of these vessels is crucial to prevent complications such as intravascular injection, vascular occlusion, haematoma formation, or tissue necrosis [25]. Pre-procedure imaging, such as ultrasound, is indispensable for mapping vascular structures, ensuring that injections are delivered at the correct depth and avoiding inadvertent damage to blood vessels.

The sensory innervation of the breast is primarily derived from the anterior and lateral branches of the intercostal nerves, which provide sensation to the breast and nipple. Care must be taken during HA filler injection to avoid damage to these nerves, particularly in patients with smaller breasts where nerves are more superficial [26,27,28,29]. Although HA fillers are generally safe and minimally invasive, inadvertent nerve injury can lead to altered sensation or discomfort post procedure. Proper technique and attention to nerve anatomy can help avoid this risk and ensure comfortable recovery for the patient.

### 1.3. Pre-Procedural Planning and Imaging

The breast is not a static structure; it changes shape and position with movement, particularly in active individuals. For younger women with more elastic skin and tissues, maintaining the breast’s natural mobility is crucial for an aesthetically pleasing result. The retromammary space is ideal for HA filler placement as it allows the filler to move naturally with the surrounding tissues, preserving the breast’s dynamic movement. Superficial injections, however, may result in a fixed, unnatural appearance that does not accommodate the breast’s natural range of motion. This is especially important for women with more active lifestyles, where preserving mobility is critical for a natural and functional outcome [30,31].

The size and shape of the breast before augmentation are key factors in determining the optimal approach for HA filler injection. Patients with larger breasts or those seeking modest enhancement may benefit from subglandular placement, where the filler can subtly increase volume without significantly altering the overall shape. Conversely, patients with smaller breasts or those seeking more significant augmentation may require submuscular placement to achieve the desired volume and contour. Additionally, breast ptosis (sagging) should be considered, as more ptotic breasts may require additional support from filler in the upper pole to restore a youthful, lifted appearance.

Pre-procedural imaging is crucial for safe HA filler breast augmentation. Ultrasound helps visualise key internal structures, such as the pectoralis fascia, retromammary space, and vascular network, ensuring precise and safe filler placement [32]. Additionally, 3D imaging systems can simulate post-procedure results, giving patients a clear preview of expected outcomes. Detailed planning, considering body type, breast volume, and aesthetic goals, ensures the appropriate filler volume and technique for natural, proportional results [33].

### 1.4. Filler Properties and Injection Techniques

Breast augmentation using HA filler is best suited for patients seeking modest breast enhancement and those who prefer non-surgical treatment. HA fillers offer a less invasive alternative to traditional implants, with lower costs and minimal recovery time. However, HA fillers are not permanent and require periodic touch-ups, which may lead to high costs in the long term. In contrast, traditional implants provide a permanent solution but come with risks and a longer recovery period. For those seeking a minimally invasive approach and are willing to undergo periodic treatments, HA filler can be a viable option, while others may prefer the permanence of implants despite their higher upfront costs.

Achieving optimal results with HA fillers depends on the following key properties: cohesiveness, elasticity, and volume retention. Cohesiveness ensures that the filler remains in place and prevents migration, while elasticity allows it to return to its original shape, preserving a natural feel and movement. These properties are particularly important in breast augmentation, as they contribute to a smooth, even result that integrates well with surrounding tissues. Volume retention is another crucial factor for long-lasting results. Fillers with a low Modified Substance (MoD) index break down more slowly, offering longer-lasting effects [34,35,36]. Additionally, HA fillers are biocompatible and integrate effectively into the body, reducing the risk of adverse reactions or complications. This makes them a preferred choice for non-surgical breast augmentation, where the goal is to enhance volume and contour while maintaining natural aesthetics. The ability to select the appropriate filler based on the patient’s specific anatomical characteristics ensures a harmonious and natural outcome [37].

The effectiveness and safety of HA filler-based breast augmentation rely on precise injection technique and the choice of appropriate cannula size and length. In this procedure, a 14-gauge, 30 cm long surgical cannula is commonly used. This cannula size allows for the safe distribution of the filler within the targeted areas, while its length ensures that the filler can be evenly distributed across a large volume of tissue, particularly in the retromammary space.

The dual-plane method is commonly preferred for this procedure, where the filler is placed beneath the pectoralis major muscle in the upper breast and into the subglandular space in the lower portion [38,39,40]. This technique creates a natural teardrop shape, enhancing the upper pole while preserving a soft, rounded lower contour. It also reduces the risk of rippling and visible lumps, making it highly effective for achieving a natural aesthetic. For patients with thin breast tissue, a submuscular approach may be chosen. This method places the filler entirely beneath the pectoralis major muscle, offering better concealment and support [11].

The key to both techniques is precise filler placement guided by pre-procedural imaging and a thorough understanding of the breast anatomy. A proper depth and angle of injection are essential to avoid complications such as vascular occlusion, asymmetry, or overfilling, which could distort the natural shape or movement of the breast. Practitioners must ensure that the filler integrates seamlessly with surrounding tissues to maintain a natural appearance and mobility.

Patients can be put under local or general anaesthesia, depending on the plane of injection or the patient’s request. Skin entry sites are usually at the anterior axillary line along the lateral pectoralis major border or lateral part of the submammary fold. Injections are made through multiple passes, leaving multiple deposits in the desired plane [11]. Continuous moulding of the injected deposits during and after the injections is crucial.

### 1.5. Post-Procedure Care and Complication Management

After HA filler-based breast augmentation, patients should avoid vigorous physical activity for several weeks and wear a supportive bra to help maintain breast shape. Unlike implants, HA fillers integrate naturally over time without the need for postoperative massage. Regular follow-up visits, typically at 1 week, 1 month, and 6 months, are essential for monitoring filler placement and complications at an early stage. Patients should be informed of the need for periodic maintenance treatments as HA fillers degrade over time.

Practitioners must be prepared to manage several risks, with intravascular injection being one of the most serious [41]. If not addressed promptly, it can lead to tissue necrosis. Ultrasound guidance during the procedure helps reduce this risk by visualising blood vessels and preventing inadvertent injection [42,43]. If an intravascular event occurs, immediate use of hyaluronidase can dissolve the filler and prevent further tissue damage [44].

Other potential complications include filler migration, asymmetry, and the formation of granulomas or nodules. Filler migration, granulomas, and nodules can be managed with manual massages or hyaluronidase, while asymmetry may require additional filler injections. In rare cases, surgery may be necessary [45,46,47,48]. Like traditional implants, HA fillers may cause capsular contractures [49]. Submuscular injection may lower the incidence of capsular contracture, as has been observed with submuscular placement of implants [50]. Capsular contracture of implants requires surgical intervention to remove or replace the implant, while capsular contracture of filler can be resolved by closed capsulotomy [11]. Regular follow-ups are crucial for early detection and timely intervention in case of complications.

## 2. Case Studies

To illustrate the effectiveness of HA fillers in breast augmentation, we present four case studies that demonstrate the versatility of this approach in achieving natural, aesthetically pleasing results. Written consent was provided, by which the patients agreed to the use and analysis of their data. This study was conducted in compliance with the principles set forth in the Declaration of Helsinki.

### 2.1. Case 1: Subtle Enhancement in a 29-Year-Old Female

**Figure 1:**
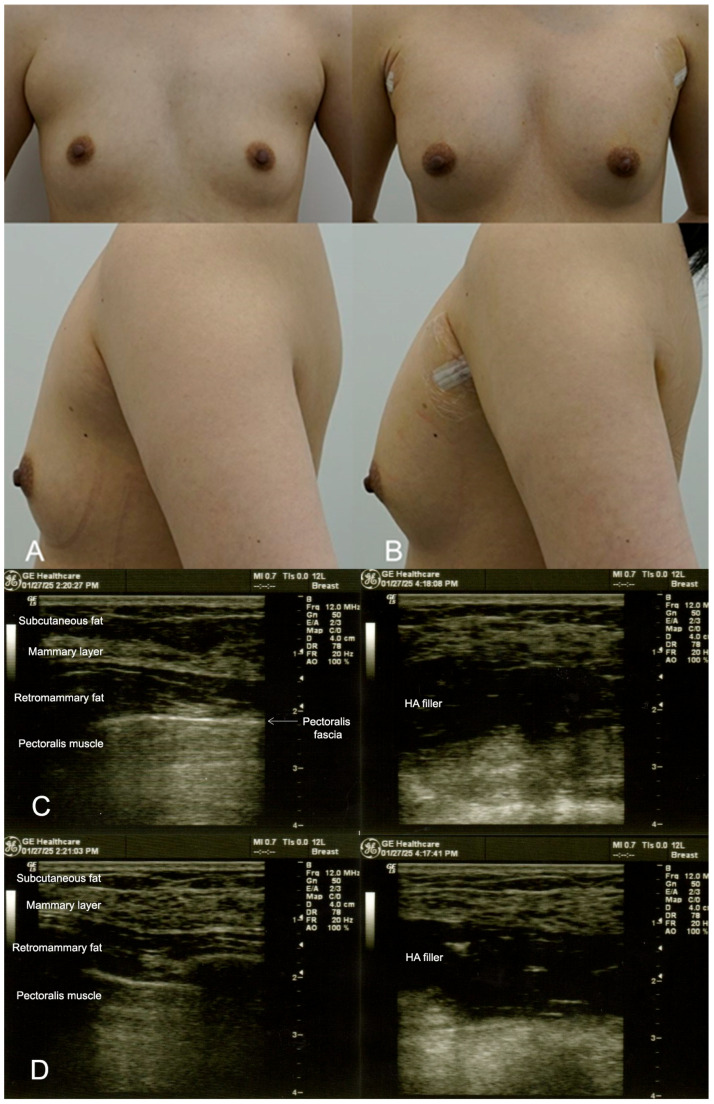
(**A**). Pre-procedure image of a 29-year-old female patient seeking subtle breast augmentation using the dual-plane technique. The patient desired a proportional increase in breast volume without undergoing surgery. (**B**). Immediate post-procedure image taken after the injection of 300 cc of HA filler (e.p.t.q. eve X, Jetema Inc., Seoul, Republic of Korea) into each breast, showing a subtle but noticeable increase in breast volume with a natural contour and smooth integration of the filler. (**C**). Ultrasound images from 9 o’clock of the breast before (**left**) and after (**right**) HA filler injection in the retromammary space, over the pectoralis fascia. An overall increase in the thickness of the breast is observed. (**D**). Ultrasound images from 6 o’clock of the breast.

This patient sought a minor increase in breast size, aiming for a more proportional figure without undergoing surgery. The dual-plane technique was utilised to inject 300 cc of HA filler (e.p.t.q. eve X, Jetema Inc., Seoul, Republic of Korea) into each breast. The filler was placed in both the subglandular and submuscular planes to create a natural teardrop shape. Ultrasound guidance was used throughout the procedure to ensure precise filler placement and to avoid vascular structures. The patient reported minimal pain post procedure and was able to resume daily activities within a few days. At the 6-month follow-up, the filler had maintained its position, and the patient expressed satisfaction with the subtle enhancement (Figure 1). However, after one year, a minor top-up was required to maintain volume.

### 2.2. Case 2: Moderate Volume Enhancement in a 35-Year-Old Patient with Thin Breast Tissue

**Figure 2:**
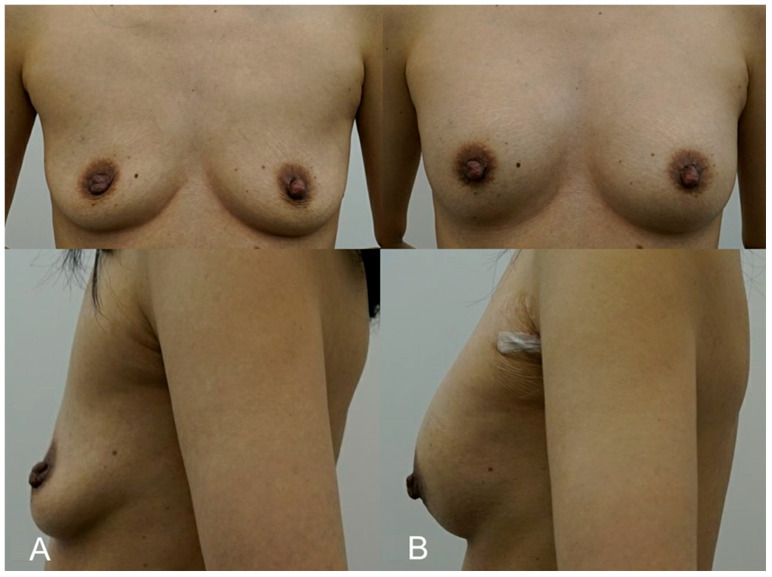
(**A**). Pre-procedure image of a 35-year-old patient with thin breast tissue, seeking a more noticeable increase in breast volume through a non-surgical approach. (**B**). Immediate post-procedure image taken after receiving 350 cc of HA filler (e.p.t.q. eve X, Jetema Inc., Seoul, Republic of Korea) into each breast using the submuscular injection technique. The image shows significant volume enhancement with no visible lumps or irregularities, demonstrating the successful placement of filler beneath the pectoralis major muscle.

A 35-year-old woman with thin breast tissue sought a more noticeable increase in breast volume while avoiding the risks associated with surgery. To achieve the desired result, 350 cc of HA filler (e.p.t.q. eve X, Jetema Inc., Seoul, Republic of Korea) was injected into each breast, with a focus on submuscular placement to provide better coverage and reduce the risk of visible lumps. Due to the patient’s anatomical limitations, the submuscular approach was deemed the most appropriate, as it allowed the filler to be concealed beneath the muscle, ensuring a smooth contour. Ultrasound imaging was used during and after the procedure to confirm the correct placement and monitor the filler. At her 6-month follow-up, the patient reported a high level of satisfaction with the results, noting that the filler had not migrated, and her breasts maintained a natural, fuller appearance (Figure 2).

### 2.3. Case 3: Subtle Enhancement for a 32-Year-Old Model

**Figure 3:**
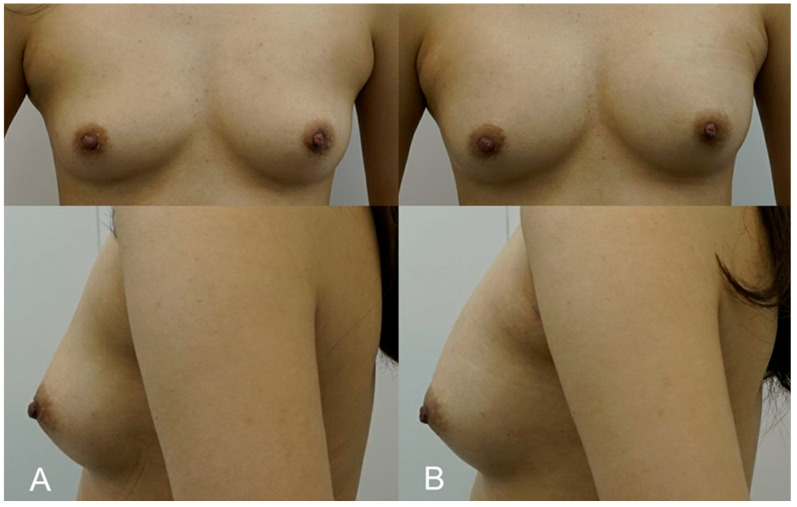
(**A**). Pre-procedure image of a 32-year-old fitness model who opted for a subtle enhancement to maintain a natural balance with her athletic physique. (**B**). Immediate post-procedure image taken after the injection of 250 cc of HA filler (e.p.t.q. eve X, Jetema Inc., Seoul, Republic of Korea) into each breast. The image illustrates a maintained natural breast contour that complements her frame, with no visible scars or complications.

A 32-year-old model desired a subtle breast enhancement that would complement her build without impeding her active lifestyle. Due to her occupation, she was keen to avoid the downtime associated with surgery. A total of 250 cc of HA filler (e.p.t.q. eve X, Jetema Inc., Seoul, Republic of Korea) was injected using the dual-plane technique to provide a modest increase in breast volume while maintaining a natural contour. The procedure allowed the patient to return to physical activity within a week. Post-procedure ultrasounds confirmed that the filler had integrated well into the breast tissue, and at the 1-year follow-up, the patient remained pleased with the result. The patient appreciated the lack of visible scarring and the ability to return to her routine quickly (Figure 3).

### 2.4. Case 4: Post-Breastfeeding Volume Restoration in a 36-Year-Old Mother

**Figure 4:**
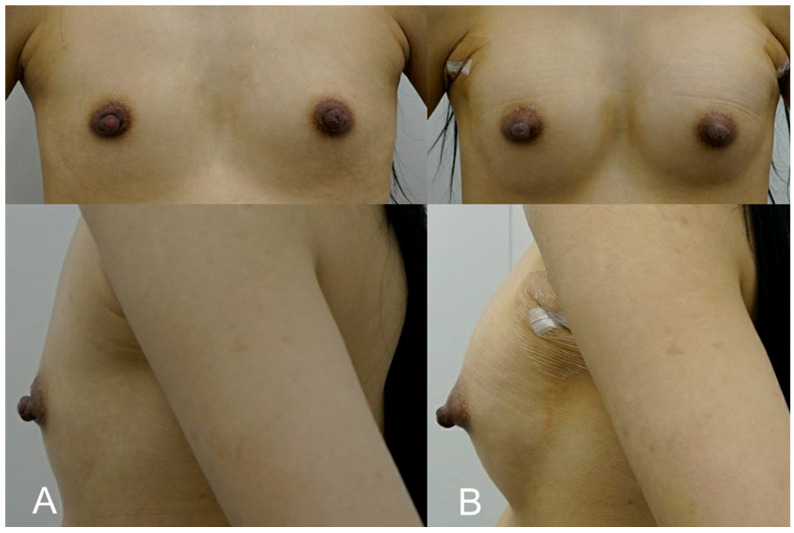
(**A**). Pre-treatment image of a 36-year-old mother of two, seeking volume restoration following breastfeeding-related changes. (**B**). Immediate post-procedure image taken after receiving 300 cc of HA filler (e.p.t.q. eve X, Jetema Inc., Seoul, Republic of Korea) in each breast, primarily targeting the upper pole. The image shows restored breast fullness and a slight lift, with a rejuvenated shape and no visible signs of surgery.

This patient, a 36-year-old mother of two, sought to restore volume and shape to her breasts following significant volume loss after breastfeeding. She was hesitant to undergo surgery but desired a noticeable improvement in breast fullness. A dual-plane approach was employed, with 300 cc of HA filler (e.p.t.q. eve X, Jetema Inc., Seoul, Republic of Korea) injected into each breast, primarily targeting the upper pole to correct volume loss and create a slight lift. The procedure restored the patient’s breasts to a fuller, more youthful shape. Mild swelling and tenderness were reported post procedure but subsided within a week. At the 6-month follow-up, the patient expressed great satisfaction, noting that her breasts felt natural and had regained their pre-pregnancy appearance without the need for invasive surgery (Figure 4).

## 3. Discussion

These four case studies highlight the versatility of HA fillers in breast augmentation, effectively addressing a range of aesthetic goals, from subtle volume enhancements to moderate restoration of breast shape. Ultrasound guidance was used in the pre-procedure phase of each case to accurately map anatomical landmarks and ensure safe filler placement in the appropriate anatomical layers. This technique is valuable in reducing the risk of complications, such as vascular injury, filler migration, and asymmetry, which can negatively affect both aesthetic outcomes and patient safety [51,52]. The selection of the appropriate filler is critical to achieving optimal results, and high-cohesiveness fillers, in general, offer better shape retention and more natural integration with the surrounding tissue, enhancing the overall aesthetic result [34,36].

One of the primary advantages of HA fillers is their minimally invasive nature, which contrasts with more traditional breast augmentation methods, such as implants. However, there are limitations and challenges to consider. One significant limitation is the temporary nature of HA fillers. Over time, these fillers degrade and require periodic touch-ups to maintain the desired aesthetic [36,53]. This presents a fundamental contrast with silicone or saline implants, which offer a more permanent solution but are associated with a range of risks, including capsular contracture and the need for surgical revision. Patients must be fully informed that although HA fillers provide immediate aesthetic results, they will require ongoing maintenance to sustain volume and contour.

Cost is another crucial factor in patient decision-making. While the upfront cost of HA filler treatment may be lower than surgery, the need for repeated treatments over time can make it a more expensive option in the long run. For patients prioritising a non-invasive approach and who are willing to undergo regular top-ups, this method may present a favourable option. However, for those who are seeking a permanent solution, traditional breast implants might be the more cost-effective and long-term choice.

As with any aesthetic intervention, the use of HA fillers in breast augmentation comes with potential risks. Filler migration, asymmetry, and the formation of granulomas remain possible complications, particularly in patients with thin breast tissue or those with highly active lifestyles [6,54]. These risks can be mitigated with careful injection techniques, the use of cohesive fillers, and vigilant post-procedure monitoring. Follow-up appointments are critical for identifying and addressing complications early, ensuring optimal outcomes. While further investigation is needed for the incidence of capsular contracture with HA fillers, it remains a complication to monitor for, particularly in patients who have previously had implants.

In terms of long-term outcomes, the patients in this study reported high satisfaction levels at both the 6-month and 1-year follow-up, with the results maintaining their shape and volume. Minor temporary swelling and tenderness were the only complications observed during the recovery period. However, it is important to recognise that HA fillers, by their nature, require regular touch-ups to sustain results, particularly for patients who received larger volumes of filler (over 300 cc). As the filler gradually degrades, some patients may notice a reduction in volume, necessitating further injections.

One important consideration, especially when using larger volumes of HA filler (i.e., over 300 cc), is the potential impact on breast imaging, particularly in terms of radiological follow-up. Changes in breast tissue due to filler injection can alter the appearance of the breast on mammograms, ultrasound, or MRI, potentially compromising the sensitivity and specificity of these imaging techniques. Filler-induced changes in the breast may mimic pathological findings, such as lumps or masses, leading to diagnostic delays or misinterpretations [55]. Therefore, it is essential for patients to inform their healthcare providers about their filler augmentation to prevent misdiagnosis. Long-term monitoring of breast health in patients who have received HA fillers is crucial to ensure that any pathological changes are promptly detected.

Ultrasound is a non-invasive and cost-effective device that offers visualisation of skin layers and injectables [56]. While the use of ultrasound guidance during the injection process helps prevent complications during the procedure, ultrasound imaging is highly operator-dependent and factors such as observer bias and interpretation variability may influence the accuracy of anatomical assessments. Training in ultrasound interpretation as well as sufficient hands-on experience is crucial [57]. According to the DERMUS group, each physicist must perform at least 300 ultrasound procedures annually to maintain the required level of competency, and standardised reports of the examination should be recorded and kept with the images [58]. Further research is needed to evaluate how HA fillers interact with radiological imaging and to develop best practices for monitoring patients post augmentation.

Finally, when large volumes of HA filler are used, it becomes necessary to reconsider the term “minimally invasive”. While HA filler-based breast augmentation is less invasive than surgical breast implants, larger volumes (exceeding 300 cc) significantly increase the complexity of the procedure and potential risks. Such treatments demand careful consideration, as they require more intensive post-procedure monitoring and the need for regular touch-ups to maintain the desired results. Future research could investigate the integration of fat or cellular injections with HA fillers to improve their efficacy in breast augmentation procedures, as transferred autologous fat demonstrates prolonged viability and requires fewer additional injections compared to HA fillers [59,60].

## 4. Conclusions

HA fillers show promise as a minimally invasive option for breast augmentation, providing moderate volume restoration and natural aesthetic results. While ultrasound guidance and careful filler selection are essential to minimise complications, the temporary nature of the fillers requires periodic maintenance treatments. Long-term follow-up is needed to fully understand the sustainability of results and the potential impact on diagnostic imaging, as fillers may obscure pathological findings. Further research is crucial to evaluate the long-term safety and efficacy of HA fillers in breast augmentation.

## Data Availability

The data are available by request to the corresponding author.

## References

[1] Jabir S., Vadodaria S., Nugent N., Sankar T.K. (2023). Breast augmentation: A cross-sectional survey of uk and irish aesthetic surgeons. Aesthet. Surg. J. Open Forum.

[2] Arian H., Alroudan D., Alkandari Q., Shuaib A. (2023). Cosmetic surgery and the diversity of cultural and ethnic perceptions of facial, breast, and gluteal aesthetics in women: A comprehensive review. Clin. Cosmet. Investig. Dermatol..

[3] Hidalgo D.A., Spector J.A. (2014). Breast augmentation. Plast. Reconstr. Surg..

[4] Swanson E. (2020). Prospective study of saline versus silicone gel implants for subpectoral breast augmentation. Plast. Reconstr. Surg. Glob. Open.

[5] Alderman A., Pusic A., Murphy D.K. (2016). Prospective analysis of primary breast augmentation on body image using the breast-q: Results from a nationwide study. Plast. Reconstr. Surg..

[6] Ishii H., Sakata K. (2014). Complications and management of breast enhancement using hyaluronic acid. Plast. Surg..

[7] Iaconisi G.N., Lunetti P., Gallo N., Cappello A.R., Fiermonte G., Dolce V., Capobianco L. (2023). Hyaluronic acid: A powerful biomolecule with wide-ranging applications-a comprehensive review. Int. J. Mol. Sci..

[8] McCleave M.J. (2010). Is breast augmentation using hyaluronic acid safe?. Aesthetic Plast. Surg..

[9] Tapsale P., Türsen B., Türsen Ü. (2022). Off label uses of hyaluronic acid fillers: A review. Dermatol. Ther..

[10] Siebert T., Chaput B., Vaysse C., Meresse T., Chavoin J.-P., Garrido I., Grolleau J.-L. (2014). The latest information on macrolane™: Its indications and restrictions. Ann. Chir. Plast. Esthet..

[11] Hedén P., Olenius M., Tengvar M. (2011). Macrolane for breast enhancement: 12-month follow-up. Plast. Reconstr. Surg..

[12] Zhang Y.L., Sun Z.S., Hong W.J., Chen Y., Zhou Y.F., Luo S.K. (2023). Biofilm formation is a risk factor for late and delayed complications of filler injection. Front. Microbiol..

[13] Park J.-H., Park J.-U., Chang H. (2021). Advances in biomaterials for breast reconstruction. Appl. Sci..

[14] Guimier E., Carson L., David B., Lambert J.M., Heery E., Malcolm R.K. (2022). Pharmacological approaches for the prevention of breast implant capsular contracture. J. Surg. Res..

[15] Jesinger R.A. (2014). Breast anatomy for the interventionalist. Tech. Vasc. Interv. Radiol..

[16] Rehnke R.D., Groening R.M., Van Buskirk E.R., Clarke J.M. (2018). Anatomy of the superficial fascia system of the breast: A comprehensive theory of breast fascial anatomy. Plast. Reconstr. Surg..

[17] Pandya S., Moore R.G. (2011). Breast development and anatomy. Clin. Obstet. Gynecol..

[18] Spear S.L., Gabriel A., Maxwell G.P., Nahabedian M.Y., Storm T. (2021). Spear’s Surgery of the Breast: Principles and Art.

[19] Gabka C.J., Bohmert H., Blondeel P.N. (2008). Plastic and Reconstructive Surgery of the Breast.

[20] Duncan A.M., Al Youha S., Joukhadar N., Konder R., Stecco C., Wheelock M.E. (2022). Anatomy of the breast fascial system: A systematic review of the literature. Plast. Reconstr. Surg..

[21] Briot N., Chagnon G., Burlet L., Gil H., Girard E., Payan Y. (2022). Experimental characterisation and modelling of breast cooper’s ligaments. Biomech. Model. Mechanobiol..

[22] Brinkman R.J., Hage J.J. (2016). Andreas vesalius’ 500th anniversary: First description of the mammary suspensory ligaments. World J. Surg..

[23] Sanchez E.R., Sanchez R., Moliver C. (2014). Anatomic relationship of the pectoralis major and minor muscles: A cadaveric study. Aesthet. Surg. J..

[24] Haley C.A., Zacchilli M.A. (2014). Pectoralis major injuries: Evaluation and treatment. Clin. Sports Med..

[25] Vegas M.R., Martina L., Segovia-Gonzalez M., Garcia-Garcia J.F., Gonzalez-Gonzalez A., Mendieta-Baro A., Nieto-Gongora C., Benito-Duque P. (2023). Vascular anatomy of the breast and its implications in the breast-sharing reconstruction technique. J. Plast. Reconstr. Aesthet. Surg..

[26] Smeele H.P., Bijkerk E., van Kuijk S.M.J., Lataster A., van der Hulst R., Tuinder S.M.H. (2022). Innervation of the female breast and nipple: A systematic review and meta-analysis of anatomical dissection studies. Plast. Reconstr. Surg..

[27] Sarhadi N.S., Shaw Dunn J., Lee F.D., Soutar D.S. (1996). An anatomical study of the nerve supply of the breast, including the nipple and areola. Br. J. Plast. Surg..

[28] Jaspars J.J., Posma A.N., van Immerseel A.A., Gittenberger-de Groot A.C. (1997). The cutaneous innervation of the female breast and nipple-areola complex: Implications for surgery. Br. J. Plast. Surg..

[29] Raman S., Daniele E., Daniele K.A., Choudhary A., Purnell C.A., Ranzer M. (2024). A scoping review of innervated breast reconstruction. Ann. Plast. Surg..

[30] Chen L.H., Ng S.P., Yu W., Zhou J., Wan K.W. (2013). A study of breast motion using non-linear dynamic fe analysis. Ergonomics.

[31] Kovacs L., Eder M., Zimmermann A., Müller D., Schuster T., Papadopulos N.A., Biemer E., Klöppel M., Machens H.-G. (2012). Three-dimensional evaluation of breast augmentation and the influence of anatomic and round implants on operative breast shape changes. Aesthet. Plast. Surg..

[32] Stöblen F., Rezai M., Kümmel S. (2010). Imaging in patients with breast implants-results of the first international breast (implant) conference 2009. Insights Imaging.

[33] Jaeger M., Randquist C., Gahm J. (2023). Anatomical breast implant assessment using ultrasound: A case series from the international breast implant check clinic. Plast. Reconstr. Surg. Glob. Open..

[34] Fundarò S.P., Salti G., Malgapo D.M.H., Innocenti S. (2022). The rheology and physicochemical characteristics of hyaluronic acid fillers: Their clinical implications. Int. J. Mol. Sci..

[35] Wongprasert P., Dreiss C.A., Murray G. (2022). Evaluating hyaluronic acid dermal fillers: A critique of current characterization methods. Dermatol. Ther..

[36] Hong G.-W., Wan J., Park Y., Yoo J., Cartier H., Garson S., Haykal D., Yi K.-H. (2024). Manufacturing process of hyaluronic acid dermal fillers. Polymers.

[37] Kenne L., Gohil S., Nilsson E.M., Karlsson A., Ericsson D., Kenne A.H., Nord L.I. (2013). Modification and cross-linking parameters in hyaluronic acid hydrogels—Definitions and analytical methods. Carbohydr. Polym..

[38] Tebbetts J.B. (2006). Dual plane breast augmentation: Optimizing implant-soft-tissue relationships in a wide range of breast types. Plast. Reconstr. Surg..

[39] Xin M., Zhang Z., Li Z. (2024). A new type of dual-plane breast augmentation: Redefining parenchyma-muscle interface in high mobile glandular ptotic breast. J. Plast. Reconstr. Aesthet. Surg..

[40] Hwang D.Y., Park S.H., Kim S.W. (2017). A modified dual-plane technique using the serratus anterior fascia in primary breast augmentation. Plast. Reconstr. Surg. Glob. Open.

[41] Murray G., Convery C., Walker L., Davies E. (2021). Guideline for the management of hyaluronic acid filler-induced vascular occlusion. J. Clin. Aesthet. Dermatol..

[42] Vasconcelos-Berg R., Izidoro J.F., Wenz F., Muller A., Navarini A.A., Sigrist R.M.S. (2023). Doppler ultrasound-guided filler injections: Useful tips to integrate ultrasound in daily practice. Aesthet. Surg. J..

[43] Lee W. (2023). Hyaluronic acid filler injection guided by doppler ultrasound. Arch. Plast. Surg..

[44] Haneke E. (2015). Managing complications of fillers: Rare and not-so-rare. J. Cutan. Aesthet. Surg..

[45] Kim H.J., Lee S.J., Lee J.H., Shin S.H., Kim S.H., Kim J.H., Suh I.S. (2020). Breast reconstruction after complications following breast augmentation with massive filler injections. Medicine.

[46] Gierej P., Woźniak-Roszkowska E., Radziszewski M., Miszczyk J., Krześniak N., Noszczyk B. (2023). Treatment of complications after minimally invasive breast augmentation with aquafilling gel. Aesthetic Plast. Surg..

[47] Jung B.K., Yun I.S., Kim Y.S., Roh T.S. (2018). Complication of aquafilling(^®^) gel injection for breast augmentation: Case report of one case and review of literature. Aesthetic Plast. Surg..

[48] Hedstrom K., Falk-Delgado A., Sackey H. (2024). Complications after breast augmentation with dermal fillers containing copolyamide: A systematic review. JPRAS Open.

[49] McCleave M.J., Grover R., Jones B.M. (2010). Breast enhancement using macrolane: A report of complications in three patients and a review of this new product. J. Plast. Reconstr. Aesthet. Surg..

[50] Young V.L., Watson M.E. (2000). Treatment of subglandular capsular contracture. Oper. Tech. Plast. Reconstr. Surg..

[51] Carloni R., Delay E., Gourari A., Quoc C.H., Tourasse C., Balleyguier C., Forme N., Goga D. (2014). Preoperative imaging prior to breast reconstruction surgery: Benchmarking bringing together radiologists and plastic surgeons. Proposed guidelines. Ann. Chir. Plast. Esthet..

[52] Schelke L.W., Decates T.S., Velthuis P.J. (2018). Ultrasound to improve the safety of hyaluronic acid filler treatments. J. Cosmet. Dermatol..

[53] Rohrich R.J., Bartlett E.L., Dayan E. (2019). Practical approach and safety of hyaluronic acid fillers. Plast. Reconstr. Surg. Glob. Open.

[54] Trignano E., Baccari M., Pili N., Serra P.L., Rubino C. (2020). Complications after breast augmentation with hyaluronic acid: A case report. Gland. Surg..

[55] Provenzano E., Pinder S.E. (2017). Modern therapies and iatrogenic changes in breast pathology. Histopathology.

[56] Ulrich J., Schwurzer-Voit M., Jenderka K.V., Voit C. (2014). Sonographic diagnostics in dermatology. J. Dtsch. Dermatol. Ges..

[57] Beiu C., Popa L.G., Bălăceanu-Gurău B., Iliescu C.A., Racoviță A., Popescu M.N., Mihai M.M. (2023). Personalization of minimally-invasive aesthetic procedures with the use of ultrasound compared to alternative imaging modalities. Diagnostics.

[58] Wortsman X., Alfageme F., Roustan G., Arias-Santiago S., Martorell A., Catalano O., di Santolo M.S., Zarchi K., Bouer M., Gonzalez C. (2016). Guidelines for performing dermatologic ultrasound examinations by the dermus group. J. Ultrasound Med..

[59] Kwiatkowska K., Krapohl B.D., Tanzella U., Ueberreiter K. (2019). Long-term clinical results and quality of life in patients undergoing autologous fat transplantation for breast augmentation using the beauli protocol. GMS Interdiscip. Plast. Reconstr. Surg. DGPW.

[60] Shuck J., Iorio M.L., Hung R., Davison S.P. (2013). Autologous fat grafting and injectable dermal fillers for human immunodeficiency virus-associated facial lipodystrophy: A comparison of safety, efficacy, and long-term treatment outcomes. Plast. Reconstr. Surg..

